# Bacteriophages in clinical samples can interfere with microbiological diagnostic tools

**DOI:** 10.1038/srep33000

**Published:** 2016-09-09

**Authors:** Maryury Brown-Jaque, Maite Muniesa, Ferran Navarro

**Affiliations:** 1 Department of Microbiology, University of Barcelona, Diagonal 645, Annex, Floor 0, 08028 Barcelona, Spain; 2 Servei de Microbiologia, Hospital de la Santa Creu i Sant Pau, Institut d’Investigació Biomèdica Sant Pau, Sant Quintí 89, 08041 Barcelona, Spain

## Abstract

Bacteriophages are viruses that infect bacteria, and they are found everywhere their bacterial hosts are present, including the human body. To explore the presence of phages in clinical samples, we assessed 65 clinical samples (blood, ascitic fluid, urine, cerebrospinal fluid, and serum). Infectious tailed phages were detected in >45% of ascitic fluid and urine samples. Three examples of phage interference with bacterial isolation were observed. Phages prevented the confluent bacterial growth required for an antibiogram assay when the inoculum was taken from an agar plate containing lysis plaques, but not when taken from a single colony in a phage-free area. In addition, bacteria were isolated directly from ascitic fluid, but not after liquid enrichment culture of the same samples, since phage propagation lysed the bacteria. Lastly, Gram-negative bacilli observed in a urine sample did not grow on agar plates due to the high densities of infectious phages in the sample.

Bacteriophages, also known as phages, are bacterial viruses that infect and multiply using the machinery of the host bacteria^1^. Phages can follow lytic or lysogenic pathways, resulting in different infection outcomes. Virulent phages only follow a lytic cycle, which begins with phage attachment to the host cell receptor, followed by injection of its nucleic acid, replication of the nucleic acid, and synthesis of capsid proteins. Capsid proteins assemble with one copy of the nucleic acid to form new viral particles, which are released by causing lysis of the host cell. In contrast, following attachment and injection of the nucleic acid into the cell, temperate phages can follow either a lytic or lysogenic cycle^2^. In the lysogenic cycle, the phage inserts its DNA into the chromosome of the bacterium and remains in a latent state (prophage) until various external conditions induce the prophage to revert to the lytic cycle.

The recognition that phages are abundant and pervasive components of many natural environments, including the human body^3^,^4^, has stimulated considerable interest in their role in microbial systems, both in regulating bacterial population density and diversity^5^ and in horizontal gene transfer^6^. In addition, there are diverse opinions on the role of phages in the homeostasis of intestinal bacterial flora and other microbiota of the human body^3^,^7^. Since many of the viruses detected in humans are phages^3^, their interactions with their cellular hosts are undeniable. Moreover, phages can mobilize genetic material by transduction, defined as phage-mediated transfer of bacterial DNA between two bacterial cells, and the use of certain antibiotics, such as beta-lactamases or quinolones, can favor and even increase genetic transduction by activating the induction of prophages^8^.

Given their abundance and ubiquity, there is a risk of phages contaminating laboratory cultures of clinical samples. Previous studies performed with enteric bacteria^9^,^10^,^11^, including studies in the area of phage therapy^12^, have indicated that the presence of phages may bias the results of enrichment cultures, where they can propagate and subsequently interfere with bacterial isolation and identification.

Some microbiological diagnosis methods applied in clinical samples are based on enriched liquid culture media (using standardized culture broths for blood, ascitic fluid, etc.), some of which (e.g. Selenite-F broth, Alkaline Peptone Water) also contain selective substances. Liquid enrichment cultures are used to increase sample volume in order to enhance analytical sensitivity, and the use of selective substances in these liquid enrichment cultures can favor the growth of the target pathogen in samples containing many other microorganisms.

Several molecular approaches based on direct detection of pathogen nucleic acid in the clinical sample have also been developed. Although these methods facilitate and accelerate diagnosis of infectious processes, some of them require preliminary steps to enrich the target microorganisms^13^. Even in molecular approaches to antibiotic susceptibility testing or epidemiological studies, it remains essential to culture pathological specimens in order to isolate and identify the causative pathogen.

It is clear that liquid enrichment cultures are prone to bias due to differential bacterial growth^9^,^11^, and we hypothesized that the interference of phages present in samples might potentially be a key factor in skewing culture results.

In clinical microbiology practice, it is not unusual to observe lysis spots consistent with phage plaques in the confluent bacterial growth on agar plates used for antibiogram assays, or even in the culture plates (Fig. 1). This observation formed the rationale for this study and prompted our hypothesis. We asked two research questions: *i*) are phages normally present in sterile human clinical samples? and *ii*) to what extent may cultured bacteria be affected by the presence of phages in the sample? Our results confirmed the presence of phages in urine and ascitic samples first analyzed directly and then following enrichment culture. Phage interference with microbiological diagnostic tools was supported by direct evidence.

## Results

### Occurrence of phages in antibiogram agar plates

**Figure 1:**
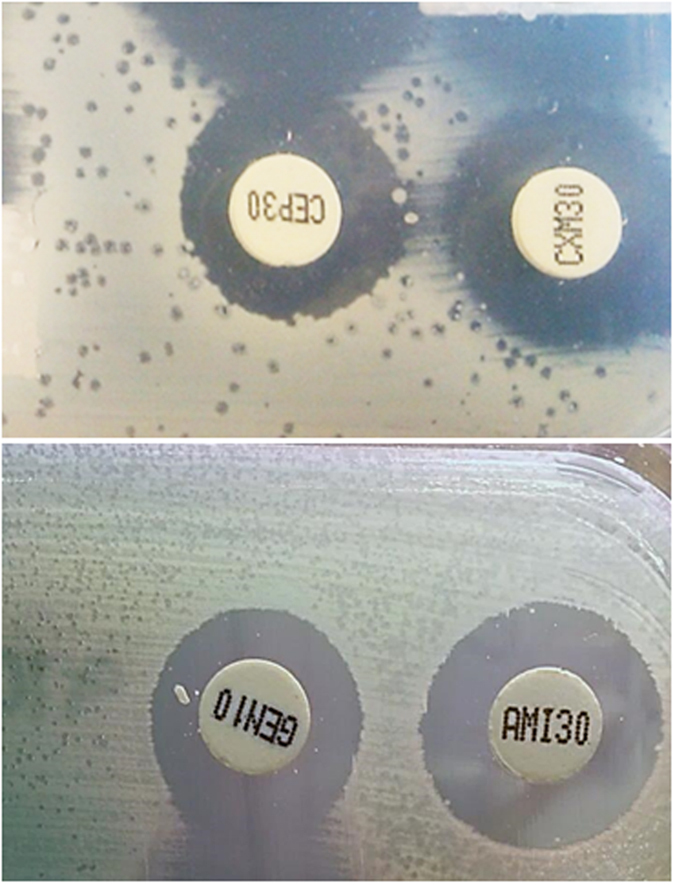
Phages on antibiogram plates. Two examples of antibiogram agar plates showing areas of confluent bacterial growth, with spots consistent with phage lysis plaques.

To confirm that the lysis spots observed in the antibiogram agar plates from urine samples (Fig. 1) were produced by phages, five plates (A to E) were selected and a 2 cm^2^ area of the plate area containing the spots was recovered and resuspended in PBS buffer. Suspensions were filtered and chloroform-treated, and then used for spot tests, phage enumeration, and electron microscopy. Four plates contained *E. coli* growth (A-D), while one corresponded to *P. aeruginosa* (E). *E. coli* WG5 was used as the host for phages in plates A-D and *P. aeruginosa*, isolated from the same plate and pure-cultured after three passages, was used as the host for phages in plate E. The spot test confirmed the presence of infectious phages in all five suspensions (Fig. 2.1), and after decimal dilution, lysis plaques were observed in the double agar layer (Fig. 2.2). All plates contained phages with *Siphoviridae* morphology (Fig. 2.3).

### Occurrence of phages in clinical samples

To further evaluate the presence of phages in normally sterile clinical samples, blood, urine, ascitic fluid, CSF and serum were analyzed. No phages were detected in blood, CSF or serum samples either by infectivity assays or electron microscopy. In contrast, 46.1% of urine samples 45.8% of ascitic fluid samples carried phages (Table 1, direct samples). In addition to these, phages were recovered from the surface of CPS agar plates inoculated with urine samples and from liquid cultures inoculated with ascitic liquids (Table 1).

The samples contained phages that infected *E. coli* WG5 and *S. sonnei* 866, as revealed using the spot test, or phage particles as observed by electron microscopy. Given that a positive spot test could be caused by factors other than phages, and that some spot tests showed weak lysis, phages were isolated from the lysis zone, diluted and enumerated by the double agar layer method, and those showing lysis plaques were confirmed.

Not all samples revealed infectious phages on *E. coli* WG5 and *S. sonnei* 866, the host strains selected for their susceptibility to a wide range of phages^14^. The *E. coli* WG5 strain was more sensitive to the phages tested and results in Table 1 were obtained with this strain. It cannot be ruled out that the use of other bacterial genera would have provided additional results, as shown for phages of *Bacteroides* in animal sera samples^15^.

Some samples analyzed directly gave a positive spot test, but phages were not subsequently observed under the electron microscope. In these cases, phages were taken from the spot test area where propagation had occurred, instead of directly from the sample, and in many instances the number of particles increased to reach the required levels (minimum of 10^7^ particles/ml) for microscopic visualization, allowing a positive confirmation. However, a few samples with a positive spot test still did not show any viral particles by microscopy of phages recovered from the lysis area (Table 1).

### Morphological types of phages in the samples

**Figure 3:**
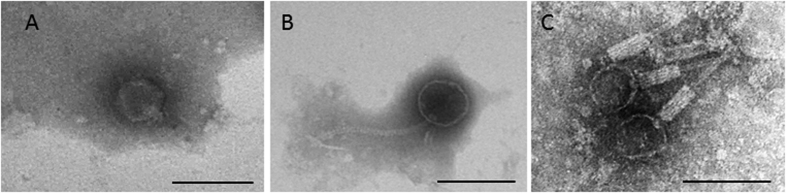
Morphological types of phages observed in samples. (**A**) *Podoviridae* from an ascitic fluid sample after propagation in WG5. (**B**) *Siphoviridae* and C: *Myoviridae* from a urine sample. (**B,C**) correspond to the same urine sample, which when processed directly showed only B and when enriched showed both. Bar 100 nm.

Twenty-five samples showed phages, the most common morphological type being *Siphoviridae* (21). Two (one urine and one ascitic fluid sample) showed *Podoviridae* morphology. A *Podoviridae* phage from one of the ascitic fluid samples was recovered after propagation in *E. coli* WG5 (Fig. 3A). Two urine samples showed phages with *Myoviridae* morphology. One of these urine samples showed *Siphoviridae* phages when analyzed directly, but after propagation on *E. coli* WG5 revealed a second *Myoviridae* phage, which was propagated in WG5 to reach the necessary concentration (Fig. 3B,C).

### Interference of phages in bacterial strain recovery

Bacteria were isolated in some of the samples. Three blood cultures showed *E. coli*, *Enterobacter cloacae* or *Staphylococcus epidermidis*, respectively. Phages were not detected in any blood samples. *E. coli* was detected in 11 urine samples and *Citrobacter koseri* and *P. aeruginosa* in another urine sample. Seven of these samples carried phages, but since no liquid culture was performed, no interference was expected.

However, we did find three examples of phage interference.

The first example corresponds to two ascitic fluid samples, both from the same patient which were processed in parallel by inoculation on agar plates and by liquid culture under aerobic and anaerobic conditions. *E. coli* was isolated from agar plates of both samples, but surprisingly, it was not detected after propagation in liquid cultures. Suspecting that phages had caused bacterial lysis during propagation in the liquid culture, but not in the agar plate, we attempted to detect phages in the samples, on the surface of the agar plate and in the supernatant of the aerobic and anaerobic liquid cultures. Phages corresponding to the *Podoviridae* and *Siphoviridae* morphological types were detected in the direct samples and in the corresponding supernatants of the aerobic liquid culture. No phages were isolated from the surface of the agar plates, probably because of the low densities of phages transferred from the samples. No phages were detected in the supernatant of anaerobic cultures either, probably because of the slow growth of the strain or the anaerobic conditions, which did not support good phage replication.

**Figure 4:**
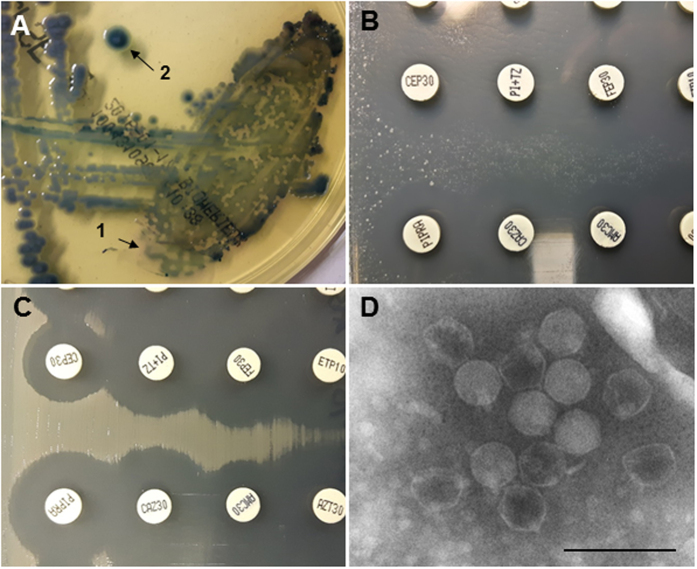
Interference of phages in antibiogram plates. (**A**) ChromID™ CPS^®^ Elite plate of a urine sample; (**B**) antibiogram agar plate inoculated directly from a colony close to the inoculation zone of this urine sample (arrow 1 in Fig. 4A); (**C**) antibiogram from an isolated colony far from the inoculation zone of this urine sample (arrow 2 in Fig. 4A). (**A,B**) show areas of confluent bacterial growth with spots consistent with phage lysis plaques but no spots were observed in (**C**). Bacteriophage of *Podoviridae* morphology isolated from (**A,B**). Bar 100 nm.

The second example of interference was in a urine sample inoculated on chromID™ CPS^®^ Elite plates (CPS plates). The confluent area showed lysis plaques consistent with the presence of phages (Fig. 4A). From this, an inoculum was taken from a colony close to the inoculation zone (Fig. 4A, arrow 1) to perform an antibiogram test. This culture was only 14 hours old and the colonies were small. The resulting antibiogram plate (Fig. 4B) showed a very poor growth of colonies, with an apparent confluent lysis. In a second test conducted 18 hours later, a single colony far from the inoculation zone (Fig. 4A, arrow 2) was taken from the CPS plate and used to inoculate a new plate, which this time yielded growth suitable for the antibiogram test (Fig. 4C). *Podoviridae*-type phages (Fig. 4D) were detected in the original urine sample and were also recovered from the surface of the first antibiogram plate (Fig. 4B), but not from the second (Fig. 4C).

**Figure 5:**
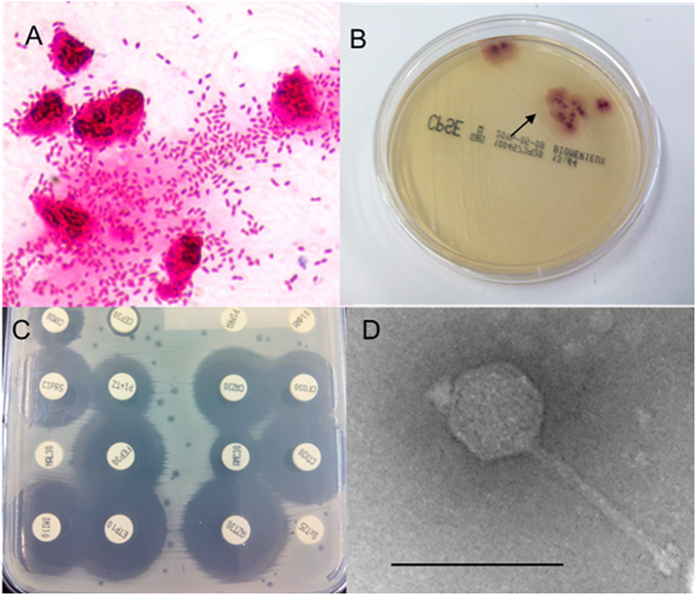
Interference of phages in bacterial isolation. (**A**) ChromID™ CPS^®^ Elite plate of a urine sample; (**B**) Gram staining of the urine sediment; (**C**) antibiogram agar plate inoculated directly from a colony (arrow in B); (**D**) bacteriophage of *Siphoviridae* morphology isolated from A. Bar 100 nm.

A third example of interference was in a urine sample obtained from a patient who had received ciprofloxacin and levofloxacin three weeks prior to the infection process, but no subsequent antimicrobial treatment before sample collection. The sediment of this urine showed 10–25 white blood cells per high-power field and abundant Gram-negative bacilli were observed by Gram staining (Fig. 5A), suggesting the presence of more than 10^5^ colony-forming units per milliliter of urine (cfu/mL). Nevertheless, only 14 *E. coli* colonies grew on the CPS plate (1.4.10^3^cfu/mL), which clearly did not correspond to the Gram stain observation. Based on the low cfu counts on the CPS plates, this sample would have been considered negative for *E. coli* were it not for the sediment analysis results. One of these colonies was used for the antibiogram agar plate, which revealed lysis plaques consistent with phages (Fig. 5B), leading us to suspect that phages in the urine sample had induced lysis of the bacteria when inoculated on the CPS plate. To confirm this, the surface of the CPS plate was washed in 3 ml PBS buffer, and the suspension was chloroform-treated and observed under the electron microscope. Observations revealed phages of *Siphoviridae* morphology (Fig. 5C) at densities higher than 10^7^ particles/ml of sample (the level required for electron microscopy observations), which were probably responsible for lysis of the bacteria growing on the CPS plate. Moreover, phages isolated from the CPS agar plate were confirmed as infectious by the spot test on the *E. coli* strain isolated from the CPS plate and pure-cultured after three passages. The phages, isolated further from the spot test area, were of the same *Siphoviridae* morphology.

## Discussion

An increasing amount of data on the diversity and abundance of phages in human bodies is becoming available. Most work in this field has focused on the digestive tract^3^,^16^,^17^ and on feces, where the first phages were isolated in the 20th century^18^,^19^, and many studies have recently been reported^20^,^21^,^22^,^23^. Nevertheless, it is also well accepted that phages are found in other biomes of the human body; for example, phages, phage DNA, or virus-like particles have been described in the skin^24^ and mouth^25^. In patients with cystic fibrosis, phages have been reported in the respiratory tract^26^, and higher concentrations of phage DNA are found in blood of cardiovascular disease patients than in healthy individuals^27^.

Phages are known to act as regulators of bacterial populations in the intestinal tract^3^,^28^, a role they might also play in other biomes within the human or animal body, although knowledge of the true extent of phages could be hampered by the inter- and intra-individual heterogeneity of the samples analyzed. It is nevertheless reasonable to assume that in the same way than bacteria can translocate from gateways in different parts of the human body, translocation might occur also with bacteriophages, as suggested a few years ago^29^. We propose phage translocation as a possible explanation for our observations in the present study. It is also possible that phages translocate alone, without accompanying bacteria, since many samples were found to carry phages without detectable bacteria. Phages can also be introduced when a sample is subjected to culture, interfering with microbiological diagnosis. This was confirmed to be the case for urine and ascitic fluid samples. CSF samples were expected to be negative because translocation should only occur there very rarely. There may be several explanations for the negative results obtained for blood and serum samples: *i*) the high protein content matrix may have disturbed microscopy observations and interfered with phage infectivity, even if using the right host strain; *ii*) phages are never present in blood or serum or only rarely; and *iii*) phage densities may have been too low to be detected by the methods used here. Nevertheless, the presence of phages in animal serum samples has been previously reported, although rarely^15^.

The bacteria and phage densities required for phage replication and subsequent destruction of the host cell^30^,^31^ can occur in some clinical samples. In our study, phages were directly observed by electron microscopy in many samples, indicating a minimum phage density of 10^7^ particles/ml of sample. Moreover, the physiological state of bacteria actively growing in a culture is optimal for phage replication^31^. Therefore, if the sample contains bacteria susceptible to infection by co-present phages, the bacterial densities achieved during enrichment could favor phage infection and replication, even when initial numbers are low. During replication, the increasing number of phages released by cell lysis can devastate a cultured bacterial population within a few hours, hence preventing isolation of the pathogen. This could account for the situation we observed in two ascitic fluid samples: *E. coli* was isolated from agar plates of both samples, but the liquid cultures tested negative for the bacteria, presumably due to lysis caused by propagation of the phages observed in the sample by electron microscopy.

Bias in the quantification of bacteria in enrichment cultures is frequently mentioned in the literature. This is typically manifested in discrepancies between the bacterial numbers detected by plating on solid medium, where phages cannot propagate so efficiently, and the counts obtained by the most probable number (MPN) method in liquid medium cultures, with surprisingly higher results obtained in agar plates than for MPN^32^. This bias cannot always be attributed to phages, which are not found in all samples, as shown in our study, and may not necessarily be able to propagate. Susceptible host bacteria might not be present, or might not be sufficiently metabolically active to allow phage replication. However, the contribution of phages is certainly a factor for consideration.

Phage interference when performing antibiograms has long been observed and documented^15^, but we noted that in some cases, the presence of phages (confirmed by electron microscopy) could even invalidate susceptibility studies. Also common in the literature are inconsistent results in microbiological diagnoses of various clinical samples (blood cultures, ascitic fluids, CSF, etc.) when a pathogen is detected and / or quantified by molecular methods (mostly PCR and qPCR) as opposed to isolation^33^,^34^. Possible explanations include prior antibiotic therapy, competition with commensal bacteria in the sample, the pathogen being in a viable but not cultivable state, or simply that the target bacteria are present in very low amounts. Based on the present study, a new additional factor for consideration is the presence of phages.

One possible scenario is that phages can lyse bacteria, preventing their isolation, although bacterial DNA may still be present in the sample. Further implications can occur when using only molecular methods based on the detection of a specific gene, or even bacterial 16SrDNA. Phages, and phage-related elements, can cause packaging of bacterial DNA and mobilize it^6^,^35^,^36^,^37^,^38^. The occurrence of free phage particles bearing this gene in a given clinical sample could consequently interfere with the molecular detection of the pathogen, because they could yield positive results of the gene detected by PCR without the bacterial pathogen actually being present. An example is Shiga toxin phages in enterohemorrhagic *E. coli*, found in abundance in human fecal samples as free viral particles in the absence of bacteria^21^. The DNA extraction methods cannot distinguish between phages and bacteria without a proper pretreatment to remove the phages^10^.

The data presented here indicate that at least some clinical samples carry phages and that these latter can bias the outcome of the clinical analysis. We suggest exploring methods for virus removal, such as filtration or the application of virucides, to reduce the presence of phages, where suspected, and contribute to more efficient recovery of bacterial isolates during microbiological diagnosis.

## Methods

### Samples

6 blood, 16 urine, 26 ascitic fluid, 7 cerebrospinal fluid (CSF), and 10 serum samples were collected from 65 patients with a potential microbial infectious disease attending the Hospital de la Santa Creu i Sant Pau (Barcelona, Spain). All samples were used after performing a conventional microbiological diagnosis and were completely anonymized. No data on patients were collected and samples were destroyed immediately after the study.

The presence of phages was evaluated, as described below, in liquid cultures of blood; directly or from agar plates for urine samples; directly or after liquid culture for ascitic samples; directly for CSF samples; directly for serums; and also from antibiogram agar plates (Table 1).

### Liquid cultures

Samples were processed according to conventional protocols for isolating pathogenic bacteria^39^. Using the BacT ⁄ALERT blood culture system (BioMérieux, Marcy l’Etoile, France), an aerobic and an anaerobic BacT⁄ALERT blood culture bottle were each inoculated with 5–10 ml of ascitic fluid or blood at the bedside. The bottles were placed in the BacT⁄ALERT instrument, incubated for five days, and processed according to the manufacturer’s instructions. Each bottle was treated independently. Cultures were not performed with serum samples, which only underwent serological testing before being evaluated for the presence of phages.

### Bacterial isolation

25 μl of ascitic fluid and CSF was inoculated in Columbia agar containing 5% sheep blood (BioMérieux), chocolate agar PolyViteX (BioMérieux) and Schaedler agar containing 5% sheep blood (BioMérieux). Cultures were incubated in 5% CO_2_ (PolyViteX and Columbia agar plates) or in an anaerobic atmosphere (Schaedler agar plate) at 35 °C for 2–4 days and examined daily for visible growth. Direct ascitic fluid and CSF were also subjected to Gram staining.

ChromID™ CPS^®^ Elite plates (BioMérieux) (CPS) were inoculated with 10 μl of urine samples, and plates were incubated aerobically at 35 °C for 24 hours. All samples previously underwent microscopic urine sediment analysis and Gram staining.

Bacteria were identified by matrix-assisted laser desorption/ionization time-of-flight mass spectrometry (MALDI-TOF MS). Susceptibility studies of microorganisms were performed by disc diffusion antibiotic susceptibility testing^40^.

### Phage purification

15 ml of blood liquid cultures, 10 ml of direct urine samples, 0.3–0.5 ml of CSF samples, 5 ml of either direct ascitic samples or liquid cultures of ascitic samples, and 5 ml of serum samples were used for phage purification. The volumes described above were filtered through low protein-binding 0.22-μm-pore-size membrane filters (Millex-GP, Millipore, Bedford, MA). When necessary, several filter units were used to filter the whole volume. If the volume was insufficient for filtration (*e.g.* CSF), it was raised to 2 ml with PBS and filtered. Filtered samples were treated with chloroform (1:10), vortexed for 2 min, and centrifuged at 16,000*xg* for 5 min. Initially, the supernatant of five ascitic liquid cultures was processed directly and after 100-fold concentration by means of protein concentrators (100 kDa Amicon Ultra centrifugal filter units, Millipore). However, we observed that the concentration step broke the phage tails and concentrated any element of a proteic nature in the sample, which disturbed electron microscopy observations. Therefore, the remaining samples were analyzed without concentration.

### Infectivity of phages present in the samples

Laboratory strains of *E. coli* WG5 (ATCC 700078) and a clinical isolate *Shigella sonnei* 866^14^ were used as hosts for bacteriophage propagation. One isolate of *Pseudomonas aeruginosa* from one antibiogram plate and one *E. coli* isolate from a CPS agar plate were also used in this study as host strains for bacteriophage propagation.

Phage propagation was performed in solid culture (by a spot test or enumeration by the double agar layer technique) or liquid culture. For the spot test, 1 ml of target bacteria at OD_600_ of 0.3 was mixed with Luria Bertrani (LB) semi-solid agar (0.7% agar), poured onto LB agar plates, and solidified at room temperature. 20 μl of phage suspension was spotted onto each of the plate surfaces, which were inspected for lysis zones after overnight incubation at 37 °C. Phage enumeration was performed by the double agar layer method as previously described^1^. Liquid cultures were performed using 1 ml of host strain and 1 ml of phage suspension in 8 ml of LB broth and incubated overnight at 37 °C while gently shaking.

Phages were recovered from the lysis zones with a loop, suspended in 0.5 ml of PBS buffer, chloroform-treated, and used for electron microscopy studies. For phage suspensions obtained from liquid cultures, 2 ml of culture was filtered and chloroform-treated as described above.

### Electron microscopy studies

10 μl of phage suspensions were dropped onto copper grids with carbon-coated Formvar films and negatively stained with 2% ammonium molybdate (pH 6.8) for 1.5 min. The samples were examined in a Jeol 1010 transmission electron microscope (JEOL Inc. Peabody, MA USA) operating at 80 kV.

### Ethics

The Ethics Committee of Hospital de la Santa Creu i Sant Pau approved the research (approval number: 12/065/1350) and waived the need for consent. The samples were anonymized.

## Additional Information

**How to cite this article**: Brown-Jaque, M. *et al*. Bacteriophages in clinical samples can interfere with microbiological diagnostic tools. *Sci. Rep.* **6**, 33000; doi: 10.1038/srep33000 (2016).

## Figures and Tables

**Figure 2:**
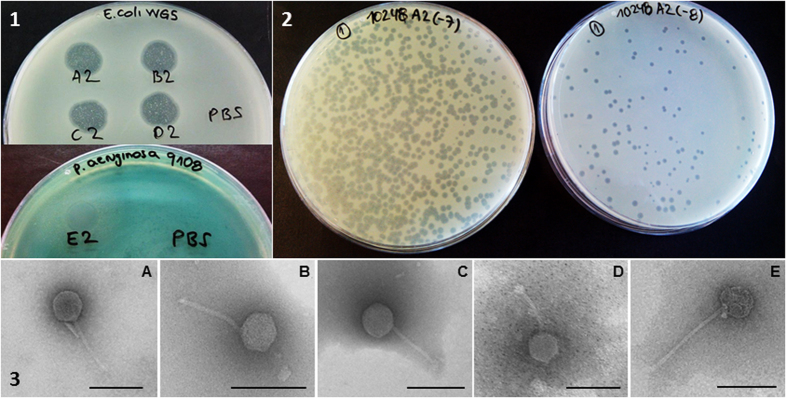
Occurrence of phages in clinical samples. Lysis plaques obtained from the suspension of antibiogram plates of urine cultures. **2.1**: Spot tests of suspensions of samples A-D in *E. coli* WG5 and E in *P. aeruginosa*. PBS control. **2.2**: Lysis plaques observed by the double agar layer method in *E. coli* WG5 in 10^−7^ and 10^−8^ dilutions of the suspension of plate A. **2.3**: Electron micrographs of phages from plates (**A–E**). Bar 100 nm.

**Table 1:** Summary of types of samples analyzed and those testing positive for the presence of phages directly from the samples or from the agar plates or the liquid cultures where the samples were inoculated.

Type of sample	Blood (6 samples)	Urine (16 samples)		Ascitic (26 samples)		CSF (7 samples)	Serum (10 samples)
	Liquid culture	Direct	Agar plate	Direct	Liquid culture	Direct	Direct
n	6	13	5	24	5	7	10
Presence of infectious phages^a^	0	10	5	16	3	0	0
Presence of phage particles^b^	0	6	5	11	3	0	0
Positive for both techniques	—	6	5	11	3	—	—
% confirmed positive	0	46.1	100	45.8	60.0	0	0

^a^Analyzed by spot test and confirmed by the double agar layer method using host strains *E. coli* WG5.

^b^Analyzed by TEM directly from the samples or recovered from the spot test lysis area.
